# Idiopathic intracranial hypertension associated with SARS-CoV-2 infection in an adult male patient: a case report and review of the literature

**DOI:** 10.1186/s13256-024-04519-x

**Published:** 2024-04-25

**Authors:** Gashaw Solela, Addis A. Tenaw, Henok Fisseha, Abel M. Argaw, Tamirat Petros, Betelhem Mengistu

**Affiliations:** 1 Department of Internal Medicine, Yekatit 12 Hospital Medical College, Addis Ababa, Ethiopia; 2 Department of Internal Medicine, St. Paul’s Hospital Millennium Medical College, Addis Ababa, Ethiopia; 3 Addis Ababa City Administration Health Bureau, Addis Ababa, Ethiopia

**Keywords:** Idiopathic intracranial hypertension, COVID-19, SARS-CoV-2 infection

## Abstract

**Background:**

Headache is a frequent symptom in coronavirus disease 2019 (COVID-19) patients, and idiopathic intracranial hypertension (pseudotumor cerebri) has been reported among patients who underwent lumbar puncture for persistent headaches.

**Case presentation:**

A 45-year-old black man presented with dyspnea, cough, fever and headache for 05 days followed by blurring of vision associated with worsening of the headache. Physical examination was significant for tachypnea and oxygen desaturation and there were no abnormal neurologic findings. He tested positive for SARS-CoV-2 with nasopharyngeal swab PCR. His CSF opening pressure appeared high with normal CSF analysis and brain magnetic resonance imaging (MRI) revealed prominent subarachnoid space around the optic nerves and bilateral papilledema. He had significant improvement with medical therapy alone.

**Conclusion:**

Idiopathic intracranial hypertension (IIH) may occur in association with SARS-CoV-2 infection and should be considered when making a differential diagnosis for headache and blurring of vision. COVID-19 may play a role in the development of intracranial hypertension, even in the absence of known risk factors. Early diagnosis and treatment of IIH has paramount importance to prevent vision loss and other morbidities.

## Background

Coronavirus disease 2019 (COVID-19), caused by severe acute respiratory syndrome coronavirus type 2 (SARS-CoV-2), has been the main global public health concern since March 2020 [1]. Although respiratory conditions are the predominating manifestations of COVID-19, neurological disorders are also becoming more frequently reported. Well known neurologic disorders reported in COVID-19 include encephalitis, encephalopathy, cerebrovascular illness, and Guillain-Barré syndrome (2).

Patients with COVID-19 frequently complain of headaches, and idiopathic intracranial hypertension (IIH), also called as pseudotumor cerebri has been observed in those who had lumbar puncture for persistent headaches (3). Patients with various neurologic conditions associated with COVID-19 have also been reported to have elevated intracranial pressure, usually in a mild form (4).

Cases reports of IIH associated with COVID-19 have been rare and they are described mainly in children and limited number of adult female patients. To the best of our knowledge, this is the first adult male patient who developed idiopathic intracranial hypertension associated with COVID-19.

## Case presentation

A 45-year-old black Ethiopian man presented to our COVID-19 isolation center with intermittent dry cough and dyspnea for 05 days associated with new onset mild holocranial headache, low grade fever, myalgia and arthralgia. One day after his admission to the center, he started to develop blurring of vision associated with worsening of the headache. He also had one episode of projectile vomiting of ingested matter. He had no known chronic medical illnesses and no history of drug intake including vitamin A derivatives and tetracycline before the onset of the aforementioned symptoms.

Physical examination revealed blood pressure of 127/91 mmHg, pulse rate of 98 beats per minute, respiratory rate of 28 breaths per minute, oxygen saturation of 86% without oxygen and 92% with 2 L/min intranasal oxygen and body mass index (BMI) of 22.8 kg/m^2^. He had bilateral coarse crepitation over his lower lung fields. He was conscious with Glasgow Coma Scale (GCS) of 15/15 and all the cranial nerves were intact with normal visual acuity (20/20) and visual fields. Meningeal signs were negative and there were no sensory or motor deficits.

Upon investigations, he tested positive for SARS-CoV-2 with nasopharyngeal swab polymerase chain reaction (PCR) and he had mild leukocytosis with left shift and lymphopenia. Lumbar puncture was performed after doing brain magnetic resonance imaging (MRI) and cerebrospinal fluid (CSF) opening pressure appeared high, though it was not measured. The other laboratory results were non-remarkable (Table 1).

**Table 1:** Summary of laboratory results

Laboratory test	Units	Results	Reference ranges
*CBC*			
WBC	Cells/mm^3^	12,500	4–10 × 10^3^
Neutrophil	%	95	50–70
Lymphocyte	%	2.8	20–40
Hemoglobin		16 g/dl	12–16
Platelet	132	150–450 × 10^3^	
Creatinine	mg/dL	0.96	0.7–1.3
Aspartate aminotransferase	U/L	29.7	< 40
Alanine aminotransferase	U/L	21.4	< 40
INR		1.1	0.8–1.2
Sodium	mmol/L	136	135–145
Potassium	mmol/L	4.08	3.5–5.5
Chlorine	mmol/L	102	96–106
Magnesium	mmol/L	1.0	0.85–1.1
HbA1C	%	5.5	< 5.7
*Cerebrospinal fluid analysis*			
Opening pressure		Appeared high	
Appearance		Crystal clear	
Cell count	cells/mm^3^	3	0–5
Lymphocytes	%	100	
Protein	mg/dL	16	15–40
Glucose	mg/dL	65	50–80
AFB/Gram stain		Negative	
PCR for SARS-CoV-2		Positive	

*CBC* Complete blood count, *WBC* White blood count, *INR* International normalized ratio, *HbA1c* Glycated hemoglobin, *AFB* Acid fast bacilli, *PCR* Polymerase chain reaction, *SARS-CoV-2* Severe acute respiratory syndrome coronavirus type 2

**Fig. 1:**
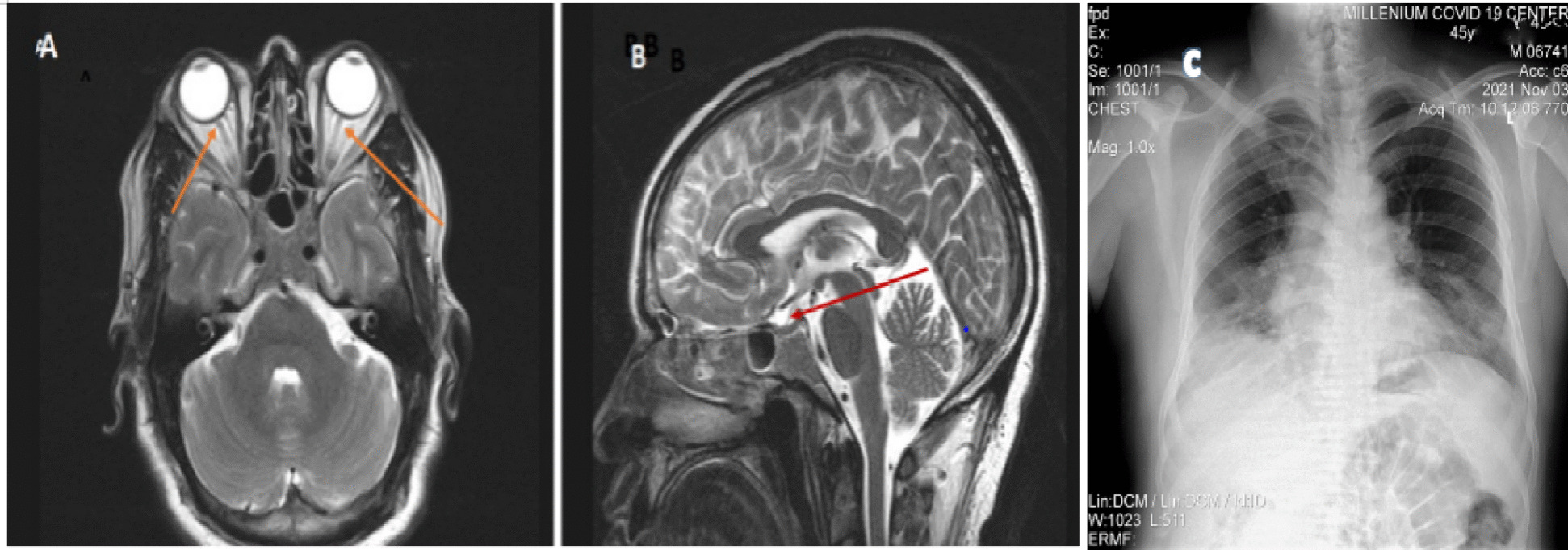
T2 axial and sagittal brain MRI scan showed prominent subarachnoid space around the optic nerves (**A**) and flattening of pituitary gland (**B**). Chest X-ray showed bilateral ground glass opacities mainly in the middle and lower lung zones with right side blunted costophrenic angle (**C**)

Brain MRI revealed prominent subarachnoid space around the optic nerves and bilateral papilledema, but didn’t show any mass lesion, hemorrhage, or cerebrovascular lesion and brain magnetic resonance venography (MRV) was normal (Fig. 1 A and B). Chest x-ray revealed bilateral ground glass opacities mainly in the middle and lower lung zones with right side blunted costophrenic angle which was suggestive of COVID-19 pneumonia (Fig. 1C).

The patient initially received supportive therapies for severe COVID-19 infection including IV antibiotics, dexamethasone 6 mg IV daily, intranasal oxygen, and prophylactic dose of unfractionated heparin. Acetazolamide 250 mg three times daily was added after establishing the diagnosis of idiopathic intracranial hypertension. He was then followed clinically if he could have an indication for ventriculoperitoneal shunt but his headache and blurring of vision improved and he did not require any surgical intervention. He was finally discharged after 10 days of inpatient supportive medical treatment with significant improvement except occasional cough and mild headache. He did not have any complaint upon reevaluation on the second week of discharge and the acetazolamide was discontinued. He was then doing well throughout his follow up over 6 months in the outpatient department.

## Discussion

Coronavirus disease 2019 (COVID-19, caused by severe acute respiratory syndrome coronavirus 2 (SARS-CoV-2), is clinically characterized by fever, myalgia, diarrhea, and respiratory illness (5). However, a number of neurological manifestations have been linked to COVID-19 in the literature, which can be divided into peripheral nervous system (PNS) manifestations like hyposmia/anosmia, hypogeusia/ageusia, myalgia, and Guillain–Barre syndrome and central nervous system (CNS) manifestations like headache, dizziness, impaired consciousness, acute cerebrovascular disease, and seizure disorders (6, 7).

Only a small number of cases of idiopathic intracranial hypertension (IIH) linked to COVID-19 have been reported, the majority of which affected children (8–10). In one study made on 58 patients with distinct neurological conditions associated with COVID-19, cerebrospinal fluid (CSF) analysis revealed that CSF pressure was elevated in one-third of COVID-19 patients with different neurological conditions and all of these patients presented early with intracranial hypertension and none of them developed loss of vision (11). In line with the results of this study, our patient appeared to have high CSF opening pressure, though not measured; indicating high intracranial hypertension and he had blurring of vision which subsequently improved with acetazolamide.

Idiopathic intracranial hypertension (IIH) is still exceedingly uncommon with 12 to 20 cases per 100,000 persons per year and the main risk factors are obesity, female gender, and reproductive age (13). The idea that obesity is an inflammatory illness is growing and markers of inflammation were found in the CSF of individuals with idiopathic IHT (13). A case series of eight adult patients described an association between IIH and COVID-19 (14). All of these patients were women; many of them were obese; and most of them improved with medical therapy alone (Table 2). Unlike the patients described in this case series, our patient was male and non-obese, but similar to most of these cases, he improved with medical therapy alone.

**Table 2:** Summary of Cases with Idiopathic Intracranial Hypertension (Pseudotumor Cerebri Syndrome) With COVID-19^14^

Age/Sex	Main complaints	BMI (kg/m^2^)	Diagnostic modality	Treatment	Outcome
22/F	Headache, blurring of vision and flu like symptoms	35.2	MRI, LP	Acetazolamide and lumbar drain	Improved
30/F	Flu like symptoms and headache	27.5	MRI, MRV	Supportive care	Improved
34/F	Flu like symptoms and blurring of vision	28.3	MRI, LP	Acetazolamide	Improved
36/F	Headache, respiratory symptoms and blurring of vision	28.2	MRI, MRV, LP	Acetazolamide	Improved
25/F	Respiratory symptoms and blurring of vision, headache	40.7	MRI, LP	Initially with acetazolamide, and VPS but didn’t improve	Improved with optic nerve sheath fenestration
51/F	Headache, cough, blurring off vision	31.9	MRI, MRV, LP	Acetazolamide, and VPS	Improved
33/F	Pharyngitis, fever, headache	33.8	MRI, MRV, LP	Acetazolamide and topiramate	Improved

*VPS* Ventriculoperitoneal shunt, *MRI* Magnetic resonance imaging, *MRV* Magnetic resonance venography

The cause of IIH in COVID-19 is uncertain and debatable. According to one study, increased CSF pressure is linked to high levels of neurofilament light chain proteins, which may be an indication of an active and exaggerated inflammatory process (11). Cerebral venous sinus thrombosis (CVT), which has been observed in COVID-19 patients, is another mechanism linked to IIH (12).

There were two case reports of middle aged female patients who had idiopathic intracranial hypertension and visual loss associated with COVID-19. The first one was a 40-year-old obese female patient, who presented with headache, bilateral optic disc edema, and visual loss, which occurred two weeks after making the diagnosis of COVID-19. Her CSF opening pressure was 410 mmH_2_O, and cranial imaging was normal. Visual loss improved after initiation of accetazolamide 250 mg three times daily (15). The second case was a 49-year-old non-obese woman, who presented with headache and vision loss and found to have COVID-19. Upon further work ups, she was diagnosed to have COVID-19 related IIH, after which she treated with mannitol infusion and oral acetazolamide 250 mg twice daily and then the headache and visual loss got improved. However, she presented again with acute vision loss, which was managed by endoscopic optic nerve fenestration surgery and then she had a good recovery (16). Our patient had similar clinical manifestations with the aforementioned cases, though he was male by gender and he did not have severe degree of visual impairment. Besides, he had remarkable improvement with medical treatment (acetazolamide) alone like that of the first case (16).

Our patient was male and had none of the aforementioned risk factors for IIH like obesity and CVT implying that SARS-CoV-2 infection could be the sole culprit in the development of IIH. To the best of our knowledge, all of the cases reported to have IIH associated with COVID-19 were women and most of them were obese and we reported the first male patient with a case of idiopathic intracranial hypertension (IIH) following COVID-19 infection.

## Conclusion

Idiopathic intracranial hypertension (IIH) may occur in association with SARS-CoV-2 infection and should be kept in mind when making a differential diagnosis for headache and blurring of vision. COVID-19 may play a role in the development of intracranial hypertension, even in the absence of known risk factors. Early diagnosis and treatment of IIH has paramount importance to prevent vision loss and other morbidities.

## Data Availability

Data supporting this case report will be available with the corresponding author upon reasonable request.
